# Post-marketing safety surveillance of pneumococcal vaccines: a real-world pharmacovigilance study using the U.S. vaccine adverse event reporting system (VAERS) database

**DOI:** 10.3389/fcimb.2025.1635180

**Published:** 2025-08-26

**Authors:** Xinkuo Zheng, Meishen Liu, Aili Ding, Shihong Zhang, Ling Wu, Fanli Kong, Weijia Sun, Yuchen Peng, Yalin Xi

**Affiliations:** ^1^ Department of Pharmacy, Central Hospital of Dalian University of Technology, Dalian, China; ^2^ Department of Pharmacy, The Second Affiliated Hospital of Dalian Medical University, Dalian, China

**Keywords:** pneumococcal vaccines, pharmacovigilance, post-marketing safety surveillance, vaccine adverse event reporting system, adverse events, safety

## Abstract

**Background:**

Pneumococcal vaccines have been utilized in the United States for decades with extensive clinical safety records. However, comprehensive post-marketing pharmacovigilance evaluations for all available types remain lacking. This study aimed to assess adverse events following immunization (AEFI) using the VAERS database and analyze potential associations between adverse events (AEs) and vaccine administration based on VAERS data.

**Methods:**

We retrieved all AEs associated with pneumococcal vaccines recorded in the VAERS database from 1990 through March 2025. Descriptive analyses were conducted to summarize the demographics, clinical characteristics, and vaccination profiles of reported cases. Disproportionality analysis was performed to detect potential safety signals between AEs and vaccine administration.

**Results:**

The VAERS database documented 157,244 individuals receiving pneumococcal vaccines, with 158,778 doses administered, capturing 632,481 AE reports following vaccination during the study period. Females showed higher AE reporting rates (54.29%) compared to males (36.88%), with the majority of cases (38.20%) occurring in individuals aged < 18 years. Complete recovery (44.20%) and hospitalization (14.94%) were the most common outcomes. Most AEs (77.11%) occurred within 0–30 days post-vaccination (median onset: 0 day). Pneumococcal polysaccharide vaccine (PPSV, 48.92%) and 13-valent pneumococcal conjugate vaccine (PCV13, 27.57%) constituted the predominant vaccine types. Disproportionality analysis identified 929 positive AE signals across 24 system organ classes (SOCs), with injection site erythema [reporting odds ratio (ROR) = 4.24], injection site swelling (ROR = 4.19), and injection site pain (ROR = 2.75) being the most frequent. Designated Medical Event (DME) screening revealed erythema multiforme (n = 398) and product contamination microbial (ROR = 11.25) as key safety signals. General disorders (ROR = 1.73) and skin conditions (ROR = 1.69) were the predominant SOC categories.

**Conclusions:**

This post-marketing surveillance has revealed predominantly non-serious AEs, with most adverse events clustered within 30 days post-vaccination. These observations reinforce the established safety profile of pneumococcal vaccines while emphasizing temporal risk patterns to guide post-vaccination monitoring protocols and risk-benefit evaluations.

## 1 Introduction

Pneumococci (also known as Streptococcus pneumoniae) infections remain a leading cause of global mortality and morbidity across all age groups (GBD 2019 Antimicrobial Resistance Collaborators, 2022; Narciso et al., 2025). This pathogen is responsible for various clinical manifestations ranging from less severe respiratory infections (including otitis media and sinusitis) to serious and potentially fatal invasive pneumococcal diseases (IPDs), encompassing pneumonia (with or without septicemia) and meningitis (Lansbury et al., 2023; Albrich et al., 2025). Epidemiological data from 2016 revealed that pneumococcal infections accounted for approximately 1.18 million deaths worldwide (GBD 2016 Lower Respiratory Infections Collaborators, 2018). Subsequent analyses of bacterial-associated mortality in 2019 identified Pneumococci as the predominant pathogen causing fatal outcomes in pediatric populations (GBD 2019 Antimicrobial Resistance Collaborators, 2022). Recognizing this significant public health challenge, the World Health Organization designated pneumococcus among 12 priority pathogens requiring urgent development of novel antimicrobial agents in their 2017 report (World Health Organization, 2017). The rising incidence of antimicrobial-resistant bacterial variants has led to significant reductions in the clinical effectiveness of existing antibiotic regimens, necessitating the development of novel treatment approaches (Antimicrobial Resistance Collaborators, 2022). Concurrently, large-scale implementation of vaccination programs has been identified as an essential component in addressing this global health challenge (Tricarico et al., 2017).

Despite extensive implementation of pneumococcal vaccination programs in the United States, provisional 2022 surveillance data indicate an IPD incidence rate of 8.3 cases per 100,000 population, with a corresponding mortality rate of 0.9 deaths per 100,000 population (Centers for Disease Control and Prevention, 2022; Washington State Department of Health, 2024). Worldwide, this pathogen accounts for an estimated 300,000 annual deaths among children younger than five years of age (Wahl et al., 2018). In the United States, the pneumococcal vaccine program evolved through sequential introductions of conjugate vaccines: a 7-valent formulation (PCV7, Prevnar^®^) received FDA approval in 2000 for pediatric immunization, followed by a 13-valent version (PCV13, Prevnar13^®^) in 2010 with expanded indications for children and adults 50 years or older (adult indication withdrawn in 2022). Subsequent developments included a 15-valent conjugate vaccine (PCV15, Vaxneuvance^®^) in 2021 and a 20-valent formulation (PCV20, Prevnar20^®^) in 2021, both licensed for adults 18 years or older. The 23-valent polysaccharide vaccine (PPSV23, Pneumovax23^®^), approved in 1983, remains indicated for high-risk individuals 2 years or older and all adults 65 years or older (Malik et al., 2025). Current Advisory Committee on Immunization Practices (ACIP) recommendations stratify usage: PCV13 is maintained for children under 5 years and immunocompromised adults 19 years or older; PCV15 requires co-administration with PPSV23 in adults 65 years or older, whereas PCV20 serves as a single-dose regimen for this age group (Kobayashi et al., 2022; Kobayashi et al., 2023).

Vigilance regarding immunization-associated adverse effects remains imperative in vaccine safety monitoring. An AEFI, defined by the Council for International Organizations of Medical Sciences (CIOMS) as any medically unfavorable incident temporally associated with vaccination without established causality, represents a critical parameter in pharmacovigilance systems. This operational definition underscores the importance of temporal association rather than confirmed causation in initial safety assessments (Council for International Organizations of Medical Sciences (CIOMS) and World Health Organization (WHO), 2012; Puliyel and Naik, 2018). The extensive global deployment of pneumococcal vaccines over three decades has resulted in widespread immunization across diverse populations, particularly pediatric cohorts and high-risk subgroups. Continuous safety monitoring remains crucial given the substantial vaccine exposure in these vulnerable groups (Wang et al., 2025). Current pharmacovigilance data demonstrate well-established safety profiles in adolescent and adult populations receiving these immunizations. The most frequently documented adverse reactions to pneumococcal immunization include localized injection site pain, erythematous reactions, and febrile responses. Post-marketing surveillance data have revealed an expanding spectrum of vaccine-associated adverse effects, encompassing severe manifestations such as respiratory distress, exacerbation of pre-existing conditions, persistent cough, and rare fatal outcomes (Oliveira et al., 2025; Wang et al., 2025).

The VAERS, a national passive surveillance system encompassing the entire U.S. population, provides critical infrastructure for pharmacovigilance (Centers for Disease Control and Prevention, 2003). Given its population-level coverage, this system enables near real-time detection of potential vaccine safety issues through identification of disproportionate adverse event reporting patterns (Iskander et al., 2004). Safety signals in pharmacoepidemiology refer to emergent temporal associations between medical products and adverse outcomes, derived from integrated analysis of pharmacovigilance data streams. Disproportionality analysis remains the gold-standard methodology for identifying rare and idiosyncratic adverse drug reactions requiring urgent investigation. Building upon this framework, our study employs comprehensive data mining of VAERS reports across multiple pneumococcal vaccine formulations to systematically characterize post-vaccination safety profiles, thereby establishing evidence-based risk stratification to inform clinical vaccination strategies.

## 2 Methods

### 2.1 Data source

This study utilized data from the United States VAERS database, a national surveillance program jointly administered by the Centers for Disease Control and Prevention (CDC) and the Food and Drug Administration (FDA) (Chen et al., 1994). Established in 1990, VAERS serves as a critical pharmacovigilance tool for monitoring post-licensure vaccine safety through four primary objectives: 1) detecting novel, atypical, or infrequent adverse events (AEs); 2) tracking frequency trends of recognized AEs; 3) identifying patient-specific risk factors associated with particular AE types; and 4) assessing safety profiles of recently approved vaccines (Centers for Disease Control and Prevention (CDC), 2024). The system employs Medical Dictionary for Regulatory Activities (MedDRA, version 27.1) Preferred Term (PT) codes for standardized AE symptom documentation, with individual case reports capturing up to five distinct clinical manifestations per VAERS identifier. This investigation classified PTs using the System Organ Class (SOC) framework (Brown, 2004).

Within the scope of this study, VAERS reports related to pneumococcal vaccines were systematically examined for the period between January 1, 1990 and March 31, 2025. The dataset encompassed all submissions for the following formulations: PNEUMO (CAPVAXIVE), PNEUMO (PNEUMOVAX), PNEUMO (PNU-IMUNE), PNEUMO (PREVNAR), PNEUMO (PREVNAR13), PNEUMO (PREVNAR20), PNEUMO (SYNFLORIX), PNEUMO (VAXNEUVANCE), and PNEUMO (NO BRAND NAME). No demographic, geographic, or clinical filters were applied to ensure inclusion of all reported adverse events regardless of age, sex, event severity, reporting source, or location. To account for potential off-label vaccination practices, reports involving age groups outside approved indication ranges were intentionally preserved for evaluation.

### 2.2 Descriptive analysis

Based on the collected reports, descriptive statistics were performed to classify AE reports associated with pneumococcal vaccines by gender, age, clinical outcome (including died, disability, hospitalized, life threatening, prolonged hospitalization, and recovered), and onset time category (within 365 days). All pneumococcal vaccines involved were stratified and statistically described by vaccine type, product name, manufacturer, and administration dose. Additionally, annual AE report counts for each pneumococcal vaccine were analyzed from 1990 through March 2025.

### 2.3 Statistical analysis

Our analytical framework employs four disproportionality analysis methods: Proportional Reporting Ratio (PRR) (Evans et al., 2001), Reporting Odds Ratio (ROR) (Rothman et al., 2004), Bayesian Confidence Propagation Neural Network (BCPNN) (Bate et al., 1998), and Multi-Item Gamma Poisson Shrinker (MGPS) (Heo and Jung, 2020). Each method demonstrates distinct strengths - PRR offers high specificity, ROR mitigates reporting bias in low-frequency events compared to PRR, BCPNN employs Bayesian inference for multi-source data integration, while MGPS enhances signal detection in rare occurrences relative to BCPNN. These complementary algorithms were integrated to counterbalance individual methodological limitations. All analytical methods employ two-by-two contingency tables (**Supplementary Table S1**), utilizing standardized computational formulas and predefined statistical thresholds (**Supplementary Table S2**). Signal strength positively correlates with vaccine-adverse event association likelihood. Following positive signal detection using the aforementioned methods, to address the issue of inflated Type I errors arising from multiple hypothesis testing across numerous vaccine-event pairs, the Benjamini-Hochberg (BH) procedure was applied to control the False Discovery Rate (FDR). A potential adverse event signal was considered statistically significant only if its BH-adjusted *p*-value (*p*-adjust) was less than or equal to the predefined FDR threshold (Q = 0.05). This correction method was applied to adjust the χ^2^ test results. Vaccine-event pairs meeting this standard (*p*-adjust ≤ 0.05) were marked as positive signals of disproportionate reporting. Analytical workflows were implemented through R statistical environment (version 4.4.2) and Microsoft Excel 2021.

### 2.4 Designated medical event list-based safety monitoring

In 2016, the European Medicines Agency (EMA) established a critical pharmacovigilance tool comprising 62 PTs categorized as DMEs (Liu et al., 2023). These medical events were identified as intrinsically severe conditions with frequent associations to medicinal products. The primary objective of this curated list is to enhance signal detection efficiency by prioritizing adverse events that warrant immediate attention, serving as an essential safeguard against oversight in pharmacovigilance activities. Our investigation specifically targeted the evaluation of significant and well-defined safety outcomes associated with pneumococcal vaccines through systematic DME list screening. The methodology involved initial signal identification using DME criteria followed by comprehensive assessment through correlation analysis with corresponding SOC categorizations.

## 3 Results

### 3.1 Demographic and clinical profile of AEs

This study analyzed 157,244 individuals who received the pneumococcal vaccine, with 632,481 AEs documented. Analysis revealed that among all patients who developed AEs, females accounted for 85,364 cases (54.29%), while males comprised 57,999 cases (36.88%). Regarding age stratification, the largest affected subgroup consisted of individuals younger than 18 years (38.20%). Among patients experiencing AEs following pneumococcal vaccination, after excluding cases with undocumented clinical outcomes, the most frequent clinical outcome was complete recovery (44.20%), followed by hospitalization attributable to the AEs (14.94%). Temporal analysis showed 77.11% patients developed AEs within 0–30 days post-vaccination. Of the documented cases, the time to onset (days) demonstrated a mean (SD) of 4.20 (22.23) with a median (min - max) of 0.00 (0 - 365) (**Table 1**).

**Table 1:** Characteristics of AE reports associated with pneumococcal vaccines from VAERS between 1990 and 2025.

Characteristics	Number of events (%)
Total	157,244 (100%)
Gender	
Female	85,364 (54.29%)
Male	57,999 (36.88%)
N/A	13,881 (8.83%)
Age (year)	
< 18	60,067 (38.20%)
18 - 64	26,600 (16.92%)
65 - 84	37,945 (24.13%)
≥ 85	2,396 (1.52%)
N/A	30,236 (19.23%)
Clinical outcome	
Died	2,837 (1.80%)
Disability	2,564 (1.63%)
Hospitalized	23,499 (14.94%)
Life threatening	3,346 (2.13%)
Prolonged hospitalization	636 (0.40%)
Recovered	69,505 (44.20%)
N/A	53,809 (34.22%)
Onset time (day)	
0 - 30	121,248 (77.11%)
31 - 60	1,289 (0.82%)
61 - 90	492 (0.31%)
91 - 120	273 (0.17%)
121 - 150	186 (0.12%)
151 - 180	145 (0.09%)
181 - 365	604 (0.38%)

AE, adverse event; VAERS, vaccine adverse event reporting system; N/A, not available.

**Figure 1:**
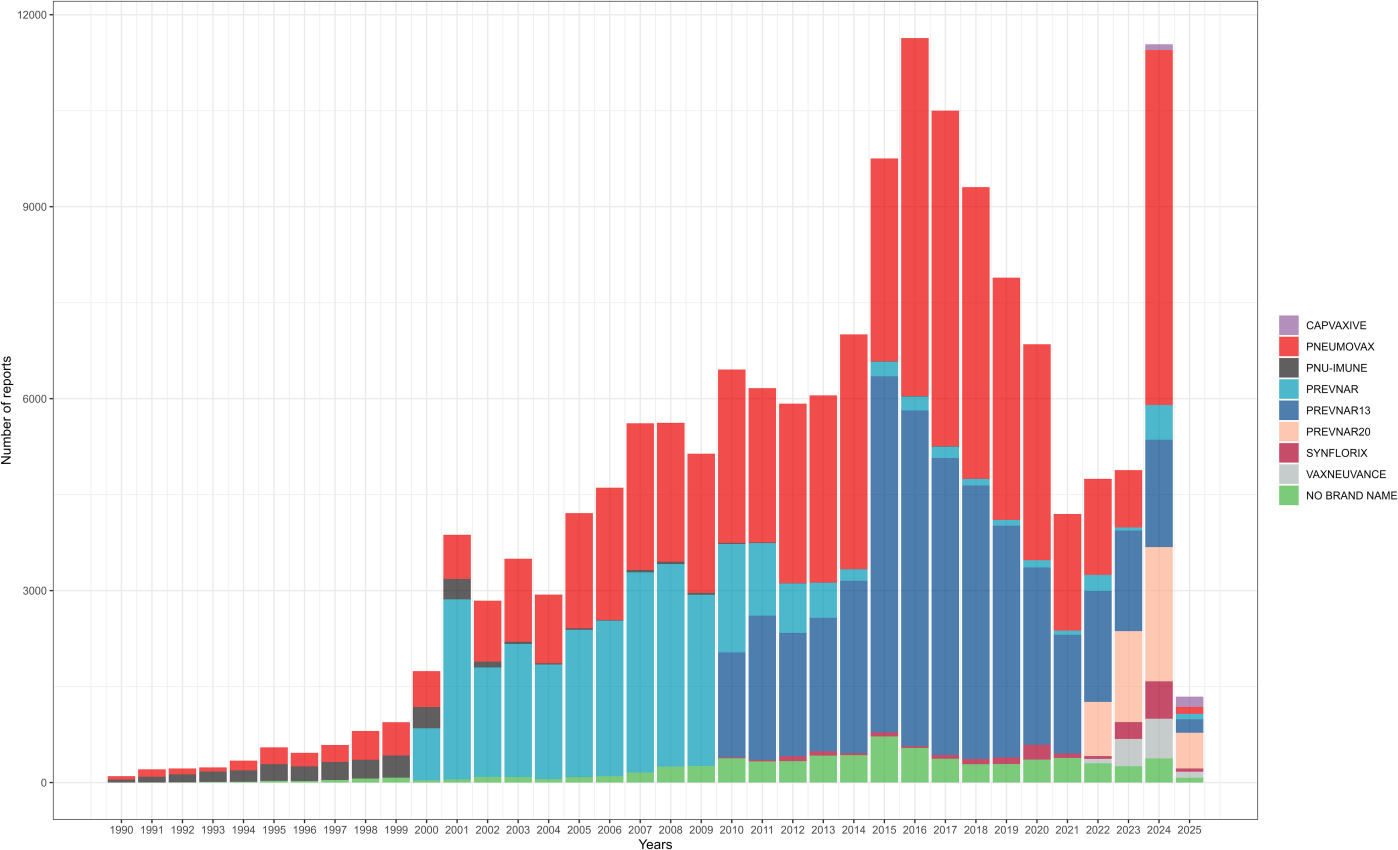
Number of reports per vaccine and per year of the pneumococcal vaccines.

Among 157,244 patients included in this study, a total of 158,778 doses of pneumococcal vaccines were administered. Nearly half of the doses were PPSV (48.92%), and among conjugate vaccines, PCV13 was the most frequently used (27.57%). The pneumococcal vaccine most frequently administered was PNEUMOVAX (42.44%), manufactured by Merck & Co., Inc. Among all pneumococcal vaccine doses, over half were produced by Pfizer/Wyeth (50.92%), followed by Merck & Co., Inc. (43.36%). Among the recorded number of doses administered, a single dose was the most common (32.27%), followed by two doses (13.49%) (**Table 2**). The annual number of reported AEs for each pneumococcal vaccine is shown in **Figure 1**.

**Table 2:** Characteristics of pneumococcal vaccines from VAERS between 1990 and 2025.

Characteristics	Number (%)
Total vaccines	158,778 (100%)
Vaccine type	
PCV7	29,160 (18.36%)
PCV10	1,791 (1.13%)
PCV13	43,773 (27.57%)
PCV15	1,212 (0.76%)
PCV20	4,926 (3.10%)
PCV21	249 (0.16%)
PPSV	77,667 (48.92%)
Vaccine name	
PNEUMO (CAPVAXIVE)	249 (0.16%)
PNEUMO (PNEUMOVAX)	67,391 (42.44%)
PNEUMO (PNU-IMUNE)	2,986 (1.88%)
PNEUMO (PREVNAR)	29,160 (18.37%)
PNEUMO (PREVNAR13)	43,773 (27.57%)
PNEUMO (PREVNAR20)	4,926 (3.10%)
PNEUMO (SYNFLORIX)	1,791 (1.13%)
PNEUMO (VAXNEUVANCE)	1,212 (0.76%)
PNEUMO (NO BRAND NAME)	7,290 (4.59%)
Vaccine manufacturer	
GLAXOSMITHKLINE BIOLOGICALS	1,791 (1.13%)
MERCK & CO. INC.	68,852 (43.36%)
PFIZER\WYETH	80,845 (50.92%)
N/A	7,290 (4.59%)
Administration dose	
1	51,220 (32.27%)
2	21,421 (13.49%)
3	9,492 (5.98%)
4	9,862 (6.21%)
5	832 (0.52%)
6	59 (0.04%)
7+	85 (0.05%)
N/A	65,807 (41.45%)

VAERS, vaccine adverse event reporting system; PCV, pneumococcal conjugate vaccine; PPSV, pneumococcal polysaccharide vaccine; N/A, not available.

### 3.2 Disproportionality analysis

**Figure 2:**
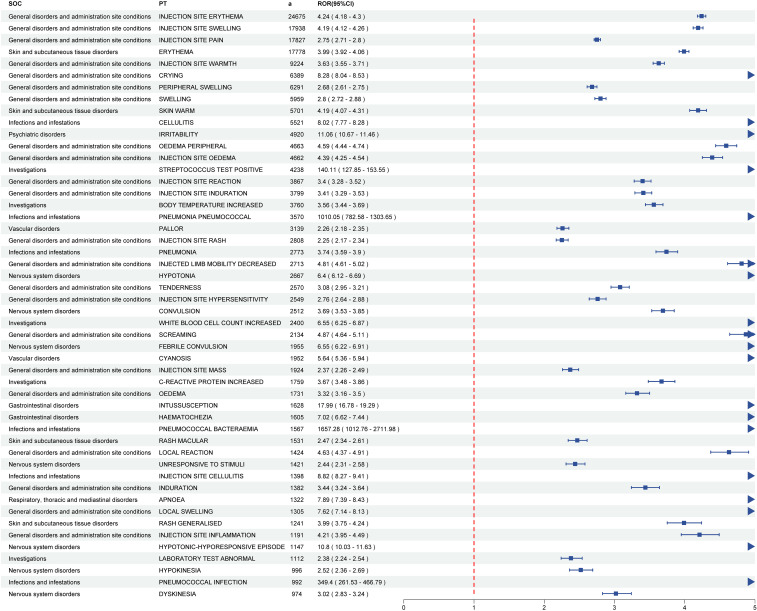
The forest plot of top fifty pneumococcal vaccine-related positive PT signals and their corresponding SOCs. PTs, preferred terms; SOC, system organ classes.

In this study, VAERS reports documented 7,740 pneumococcal vaccine-associated PTs spanning 27 SOCs. Through comprehensive analysis using four pharmacovigilance methods (ROR, PRR, BCPNN, MGPS), 929 positive PT signals were ultimately identified across 24 SOCs. The five most frequent PTs for positive AE signals were injection site erythema (a = 24,675, ROR = 4.24, PRR = 4.12, IC = 1.81, EBGM = 3.51), injection site swelling (a = 17,938, ROR = 4.19, PRR = 4.10, IC = 1.80, EBGM = 3.49), injection site pain (a = 17,827, ROR = 2.75, PRR = 2.70, IC = 1.30, EBGM = 2.47), erythema (a = 17,778, ROR = 3.99, PRR = 3.90, IC = 1.75, EBGM = 3.36), and injection site warmth (a = 9,224, ROR = 3.63, PRR = 3.59, IC = 1.65, EBGM = 3.14). The top fifty pneumococcal vaccine-related positive PT signals and their corresponding SOCs are shown in **Figure 2**; **Supplementary Table S3**. For the 929 positive PT signals, BH correction was applied. The results demonstrated that all adjusted *p*-values (*p*-adjust) were ≤ 0.05, indicating these signals remained statistically significant after multiple testing correction (**Supplementary Table S3**).

**Figure 3:**
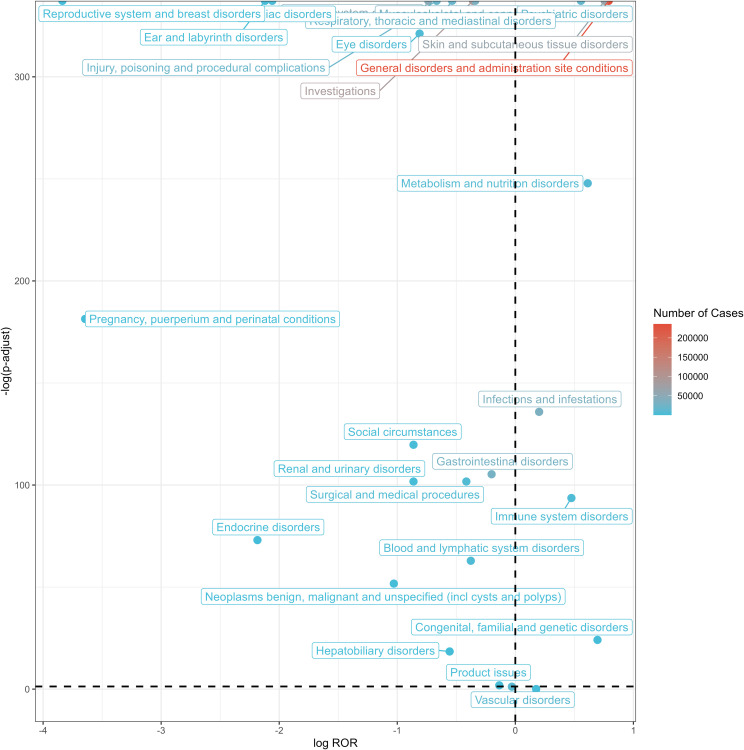
Volcano plot of SOCs corresponding to all reported AEs. SOC, system organ classes; AEs, adverse events; ROR, reporting odds ratio.

The five most frequent SOCs were general disorders and administration site conditions (a = 235,837, ROR = 1.73, PRR = 1.46, IC = 0.51, EBGM = 1.42), investigations (a = 89,777, ROR = 0.78, PRR = 0.81, IC = -0.29, EBGM = 0.82), skin and subcutaneous tissue disorders (a = 62,387, ROR = 1.69, PRR = 1.62, IC = 0.65, EBGM = 1.57), nervous system disorders (a = 46,279, ROR = 0.60, PRR = 0.63, IC = -0.63, EBGM = 0.64), and infections and infestations (a = 33,754, ROR = 1.15, PRR = 1.15, IC = 0.19, EBGM = 1.14). The relationship between effect sizes (ROR) and statistical significance for pneumococcal vaccine-associated AE signals is shown in **Figure 3**. Signal strengths of pneumococcal vaccine-related AEs at the SOC level are reported in **Table 3**.

**Table 3:** Signal strength of AE reports of pneumococcal vaccines at the SOC level.

SOC	a	ROR (95%Cl)	PRR (χ^2^)	EBGM (EBGM05)	IC (IC-2SD)
General disorders and administration site conditions	235847	1.73 (1.72, 1.74)	1.46 (42399.51)	1.42 (1.42)	0.51 (0.50)
Investigations	89777	0.78 (0.77, 0.78)	0.81 (4617.9)	0.82 (0.81)	-0.29 (-0.30)
Skin and subcutaneous tissue disorders	62387	1.69 (1.68, 1.71)	1.62 (14510.58)	1.57 (1.56)	0.65 (0.64)
Nervous system disorders	46279	0.60 (0.6, 0.61)	0.63 (10824.43)	0.64 (0.64)	-0.63 (-0.65)
Infections and infestations	33754	1.15 (1.14, 1.17)	1.15 (620.16)	1.14 (1.13)	0.19 (0.17)
Musculoskeletal and connective tissue disorders	33741	0.79 (0.78, 0.79)	0.80 (1790.32)	0.81 (0.80)	-0.31 (-0.33)
Gastrointestinal disorders	29070	0.87 (0.86, 0.88)	0.88 (479.35)	0.89 (0.88)	-0.18 (-0.19)
Respiratory, thoracic and mediastinal disorders	19559	0.69 (0.68, 0.7)	0.70 (2592.24)	0.71 (0.70)	-0.50 (-0.52)
Injury, poisoning and procedural complications	19494	0.63 (0.62, 0.64)	0.64 (4064.73)	0.65 (0.64)	-0.62 (-0.64)
Psychiatric disorders	16579	1.47 (1.45, 1.49)	1.46 (2225.58)	1.42 (1.40)	0.51 (0.48)
Vascular disorders	9811	0.98 (0.96, 1.00)	0.98 (3.49)	0.98 (0.97)	-0.03 (-0.06)
Metabolism and nutrition disorders	6789	1.53 (1.49, 1.57)	1.53 (1135.93)	1.48 (1.45)	0.57 (0.53)
Surgical and medical procedures	5723	0.75 (0.73, 0.77)	0.75 (462.99)	0.76 (0.74)	-0.4 (-0.43)
Eye disorders	4711	0.57 (0.55, 0.59)	0.57 (1473.45)	0.59 (0.57)	-0.77 (-0.81)
Blood and lymphatic system disorders	4557	0.77 (0.75, 0.8)	0.78 (284.22)	0.79 (0.77)	-0.35 (-0.39)
Immune system disorders	4298	1.39 (1.34, 1.43)	1.38 (425.72)	1.36 (1.32)	0.44 (0.39)
Cardiac disorders	3163	0.24 (0.23, 0.25)	0.24 (7516.35)	0.25 (0.25)	-1.98 (-2.03)
Social circumstances	1494	0.55 (0.52, 0.57)	0.55 (546.18)	0.56 (0.54)	-0.83 (-0.91)
Renal and urinary disorders	1284	0.55 (0.52, 0.58)	0.55 (463.10)	0.56 (0.54)	-0.83 (-0.91)
Ear and labyrinth disorders	1245	0.23 (0.22, 0.24)	0.23 (3158.26)	0.24 (0.23)	-2.05 (-2.13)
Product issues	794	0.91 (0.85, 0.98)	0.91 (6.26)	0.92 (0.86)	-0.12 (-0.23)
Hepatobiliary disorders	559	0.68 (0.63, 0.74)	0.68 (80.64)	0.69 (0.65)	-0.53 (-0.65)
Congenital, familial and genetic disorders	489	1.62 (1.48, 1.78)	1.62 (106.81)	1.57 (1.45)	0.65 (0.51)
Neoplasms benign, malignant and unspecified	454	0.49 (0.45, 0.54)	0.49 (233.08)	0.51 (0.47)	-0.98 (-1.12)
Reproductive system and breast disorders	418	0.07 (0.06, 0.07)	0.07 (5315.42)	0.07 (0.07)	-3.79 (-3.93)
Endocrine disorders	123	0.22 (0.19, 0.26)	0.22 (331.38)	0.23 (0.20)	-2.11 (-2.37)
Pregnancy, puerperium and perinatal conditions	74	0.08 (0.06, 0.10)	0.08 (830.46)	0.08 (0.07)	-3.64 (-3.98)

AE, adverse event; CI, confidence interval; SOC, System Organ Class; ROR, reporting odds ratio; PRR, proportional reporting ratio; χ^2^, Chi-squared; IC, information component; IC-2SD, the lower limit of the 95% two-sided CI of the IC; EBGM, empirical Bayesian geometric mean; EBGM05, the lower 95 two-sided CI of EBGM.

### 3.3 DME list screening

**Figure 4:**
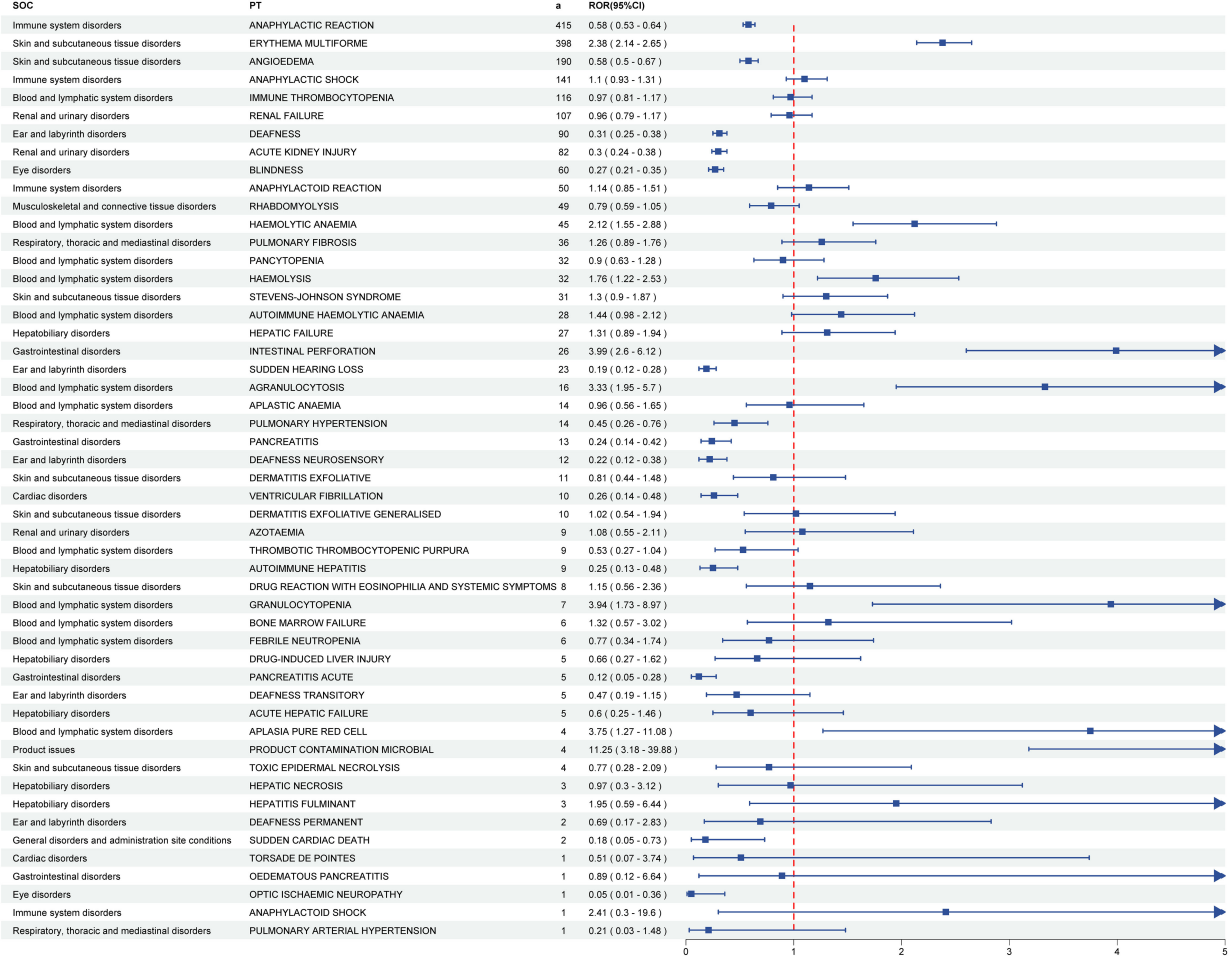
The forest plot of DME screening results for pneumococcal vaccines. DME, designated medical events. PTs, preferred terms; SOCs, system organ classes.

Developed by the EMA, the DME registry catalogues suspected AEs necessitating prioritized pharmacovigilance monitoring. Of the total detected signals, 51 DME-associated PT signals spanning 13 SOCs were identified. Subsequent screening revealed 8 positive PT signals involving 4 SOCs. Among the positive signals, the two most frequent DME signals were erythema multiforme (a = 398) and haemolytic anaemia (a = 45), classified under skin and subcutaneous tissue disorders and blood and lymphatic system disorders, respectively. The two most robust DME signals were product contamination microbial (ROR = 11.25) and intestinal perforation (ROR = 3.99), categorized under product issues and gastrointestinal disorders, respectively (**Figure 4**; **Supplementary Table S4**; **Supplementary Figure S1**).

### 3.4 Sensitivity analysis

We have noted that some data in the VAERS system are missing or not available. For example, age is missing in 19.23% of reports, dose information is missing in 41.45% of reports, and clinical outcome is missing in 34.22% of reports. To minimize the impact of these missing values, we performed sensitivity analyses. For age, dose information, and clinical outcome, we removed records containing missing data and then performed sensitivity analyses for each variable. **Table 4** presents the top 10 positive PT signals for age, dose, and clinical outcome after removing records with missing data. After comparison, although the ranking of positive PT signal strength shifted, no fundamentally different results emerged.

**Table 4:** Sensitivity analysis of positive PT signals following missing data removal.

PT	a	ROR (95%Cl)	PRR (χ^2^)	EBGM (EBGM05)	IC (IC-2SD)
Age					
INJECTION SITE ERYTHEMA	22827	3.63 (3.58 - 3.68)	3.52 (33689.15)	3.03 (3.00)	1.60 (1.58)
ERYTHEMA	16436	3.59 (3.53 - 3.65)	3.51 (24115.71)	3.03 (2.99)	1.60 (1.57)
INJECTION SITE SWELLING	16381	3.53 (3.47 - 3.59)	3.45 (23346.10)	2.99 (2.94)	1.58 (1.55)
INJECTION SITE PAIN	16151	2.35 (2.31 - 2.39)	2.31 (10484.61)	2.13 (2.10)	1.09 (1.07)
INJECTION SITE WARMTH	8652	3.10 (3.03 - 3.17)	3.07 (10032.39)	2.71 (2.66)	1.44 (1.40)
CRYING	5702	7.23 (7.01 - 7.47)	7.17 (20421.31)	5.15 (5.02)	2.37 (2.32)
PERIPHERAL SWELLING	5615	2.58 (2.51 - 2.66)	2.57 (4589.62)	2.33 (2.28)	1.22 (1.18)
SWELLING	5412	2.62 (2.55 - 2.70)	2.61 (4581.18)	2.37 (2.31)	1.24 (1.20)
SKIN WARM	5409	3.62 (3.52 - 3.73)	3.60 (8178.86)	3.09 (3.01)	1.63 (1.58)
CELLULITIS	4654	6.87 (6.63 - 7.11)	6.82 (15843.13)	4.98 (4.84)	2.32 (2.27)
Administration dose					
INJECTION SITE ERYTHEMA	15823	4.37 (4.29 - 4.44)	4.22 (34419.04)	3.82 (3.76)	1.93 (1.91)
INJECTION SITE SWELLING	10999	4.09 (4.00 - 4.17)	3.99 (21911.56)	3.64 (3.57)	1.86 (1.83)
ERYTHEMA	10854	3.89 (3.81 - 3.97)	3.81 (20037.8)	3.48 (3.42)	1.80 (1.77)
INJECTION SITE PAIN	10429	2.63 (2.57 - 2.68)	2.58 (9388.74)	2.45 (2.41)	1.29 (1.26)
INJECTION SITE WARMTH	5928	3.78 (3.67 - 3.88)	3.73 (10565.35)	3.42 (3.35)	1.78 (1.74)
CRYING	4708	9.43 (9.13 - 9.75)	9.33 (26613.31)	7.32 (7.12)	2.87 (2.82)
IRRITABILITY	3833	13.14 (12.64 - 13.65)	13.01 (29483.11)	9.32 (9.03)	3.22 (3.17)
PERIPHERAL SWELLING	3578	2.48 (2.40 - 2.57)	2.47 (2891.4)	2.35 (2.29)	1.23 (1.18)
SKIN WARM	3542	4.14 (4.00 - 4.29)	4.11 (7340.98)	3.73 (3.62)	1.90 (1.85)
SWELLING	3500	2.68 (2.59 - 2.77)	2.66 (3338.88)	2.52 (2.45)	1.33 (1.28)
Clinical outcome					
INJECTION SITE ERYTHEMA	12636	3.20 (3.14 - 3.26)	3.13 (16624.74)	2.91 (2.87)	1.54 (1.52)
ERYTHEMA	9206	3.07 (3 - 3.13)	3.02 (11313.26)	2.82 (2.77)	1.50 (1.47)
INJECTION SITE SWELLING	9047	3.11 (3.04 - 3.18)	3.06 (11401.05)	2.86 (2.80)	1.51 (1.48)
CRYING	5021	9.81 (9.5 - 10.13)	9.69 (29131.17)	7.46 (7.26)	2.90 (2.85)
INJECTION SITE WARMTH	4376	2.56 (2.48 - 2.64)	2.54 (3771.93)	2.41 (2.35)	1.27 (1.23)
IRRITABILITY	3451	10.79 (10.37 - 11.22)	10.70 (21981.65)	8.02 (7.76)	3.00 (2.95)
CELLULITIS	3118	6.23 (5.99 - 6.48)	6.19 (11124.41)	5.25 (5.08)	2.39 (2.34)
SWELLING	3068	2.21 (2.13 - 2.29)	2.20 (1868.44)	2.11 (2.05)	1.08 (1.02)
INJECTION SITE OEDEMA	3028	4.34 (4.18 - 4.51)	4.32 (6696.75)	3.87 (3.75)	1.95 (1.90)
OEDEMA PERIPHERAL	2870	4.24 (4.08 - 4.41)	4.22 (6140.17)	3.80 (3.68)	1.93 (1.87)

PT, preferred term; CI, confidence interval; ROR, reporting odds ratio; PRR, proportional reporting ratio; χ^2^, Chi-squared; IC, information component; IC-2SD, the lower limit of the 95% two-sided CI of the IC; EBGM, empirical Bayesian geometric mean; EBGM05, the lower 95 two-sided CI of EBGM.

## 4 Discussion

To our knowledge, this pharmacovigilance study is the first to comprehensively integrate safety signals across multiple pneumococcal vaccines using the U.S. VAERS database. This study assessed post-licensure pneumococcal vaccines’ safety using VAERS database disproportionality assessments and DME list monitoring. Disproportionality analysis revealed that pyrexia, injection site erythema, swelling, and pain, along with generalized erythema, constituted the most frequently reported AEs following pneumococcal vaccination. The majority of cases were non-severe, consisting primarily of self-limited localized and systemic events that mirrored pre-licensure study observations (Hurley et al., 2021; Klein et al., 2021). These results align with VAERS safety assessments of PCV13 in individuals aged ≥ 19 years (Haber et al., 2016). Among 7,740 detected PTs, 51 signals matched PTs in the DME list, with 8 positive signals comprising erythema multiforme, haemolytic anaemia, hemolysis, intestinal perforation, agranulocytosis, granulocytopenia, pure red cell aplasia, and microbial product contamination.

According to VAERS data, 81.84% of AEs following pneumococcal vaccination were classified as non-serious, while 18.16% constituted serious AEs. Among the serious AEs, 2,837 fatal cases were reported, accounting for approximately 1.79% of all AEs. An AEFI is defined as any untoward medical occurrence temporally associated with vaccination, which may either represent a true adverse reaction causally linked to the vaccine or a coincidental event unrelated to immunization. Notably, among all documented serious AEs, the 116 cases of immune system disorders represented a relatively small proportion. All four disproportionality analysis metrics (PRR, ROR, BCPNN, and MGPS) demonstrated non-significant signals for these immune-related events, indicating no statistically detectable safety concern. This finding aligns with previous reports from the three agencies: WHO, CDC, and EMA (World Health Organization, 2019; Olivieri et al., 2021; Kobayashi et al., 2025). Anaphylaxis may rarely be causally associated with vaccination. Our analysis identified only one anaphylaxis case with insufficient evidence to meet the Brighton criteria, and notably, anaphylaxis did not constitute a positive safety signal (Rüggeberg et al., 2007).

It is noteworthy that this study observed 77.11% of AE reports clustering within the 0- to 30-day window following pneumococcal vaccination. However, establishing a causal relationship between vaccination and an adverse event requires a comprehensive framework for assessment. This necessitates adherence to systematic causality assessment methods, such as those outlined in the WHO guidelines for Causality Assessment of AEFI (World Health Organization, 2021). These guidelines emphasize the integration of multiple factors, including temporal association, biological plausibility, dechallenge or rechallenge responses (where applicable), and consideration of alternative etiologies, among others. Consequently, while the observed temporal clustering is a necessary criterion for evaluating potential causality, it is not sufficient on its own. This pattern may be influenced by factors such as reporting bias or coincidental occurrence of unrelated events, and therefore cannot definitively establish causation.

The analysis of pneumococcal vaccine characteristics from VAERS reports between 1990 and 2025 reveals distinct patterns in vaccine utilization and reporting trends. The majority of reported AEs were associated with PPSV (48.92%) and PCV13 (27.57%), likely reflecting their long-standing recommendations for adults and pediatric populations, respectively (Berman-Rosa et al., 2020). In contrast, formulations such as PCV15 (0.76%) and PCV20 (3.10%) showed limited representation, possibly due to their recent introduction and phased implementation in vaccination programs. Manufacturer data highlighted the dominance of Pfizer/Wyeth (50.92%) and Merck & Co. (43.36%), aligning with their roles as primary producers of conjugate and polysaccharide vaccines. These findings emphasize the need for continued monitoring of newer vaccines as their adoption increases, while also advocating for improved documentation of administration details to enhance post-marketing surveillance accuracy.

Revaccination demonstrates a higher prevalence of injection site reactions compared to primary vaccination (Kim et al., 2020). In our dataset, dose sequence information was missing in 41.45% of pneumococcal vaccine reports, consistent with prior observations of inconsistent vaccination sequence documentation in VAERS reports across multiple vaccine types. These limitations precluded meaningful dose-stratified analysis of injection site symptoms. While pneumococcal vaccines may be administered via intramuscular (IM) or subcutaneous (SC) routes, with evidence suggesting reduced local reactogenicity for IM administration, comparative analysis of AE profiles between IM and SC routes was not feasible in this study due to insufficient documentation quality in VAERS records (Herzog, 2014; Miller et al., 2016).

A key strength of VAERS lies in its nationwide coverage and capacity for real-time reporting. This system serves as a critical tool for early identification of rare AEs, which, if novel or unexpected, may trigger targeted investigations through active surveillance platforms or epidemiological studies. However, as a passive reporting framework, VAERS inherently faces limitations in data granularity and causality assessment, necessitating cautious interpretation of observed associations. While external studies indicate that > 70% immunization-coverage was achieved with PCV7 in at least 10 countries during its active use period (World Health Organization, 2019), this population-level context cannot be obtained from the VAERS system. A critical limitation inherent to the VAERS system is the absence of denominator data—specifically, the total number of vaccine doses administered (i.e., exposure counts). This lack of exposure denominators precludes calculating true incidence rates or risks for reported adverse events. Consequently, observed patterns reflect only the distribution among submitted reports and cannot be extrapolated to estimate actual event frequency or risk in the vaccinated population. Several notable constraints of VAERS must be acknowledged, including underreporting, variable report quality (e.g., incomplete entries or factual errors), and the lack of an unvaccinated control cohort. Additionally, critical clinical or laboratory details may be absent in follow-up medical records of severe cases. These limitations substantially hinder causal attribution between vaccines and reported AEs (McNeil et al., 2014). Despite these challenges, VAERS remains valuable for initial identification of rare safety signals and emerging concerns, though such findings require confirmation through methodologically robust epidemiological investigations. Future investigations should incorporate longitudinal monitoring, treatment assessments, and integrated pharmacokinetic analyses to establish causality. Notwithstanding these constraints, our findings offer preliminary insights for subsequent research and may inform more evidence-based vaccine utilization strategies.

## 5 Conclusions

Analysis of pneumococcal vaccine-associated AEs in the VAERS database identified 929 significant PTs through disproportionality analysis. Predominant safety concerns included injection site reactions, erythema, crying, swelling, skin warm, and cellulitis. The majority of AEs occurred within the initial 30-day post-vaccination period. These findings offer critical insights for clinical safety surveillance of pneumococcal vaccines and inform decision-making by regulatory agencies and healthcare professionals regarding risk mitigation strategies.

## Data Availability

Publicly available datasets were analyzed in this study. This data can be found here: The data utilized in these analyses are publicly accessible through the VAERS dataset, available at https://vaers.hhs.gov/index.html.
