# Supplementary material for: Post-marketing safety surveillance of pneumococcal vaccines: a real-world pharmacovigilance study using the U.S. vaccine adverse event reporting system (VAERS) database
Source: Front Cell Infect Microbiol. 2025 Aug 26;15:1635180. doi: 10.3389/fcimb.2025.1635180 (PMC12417412; doi:10.3389/fcimb.2025.1635180)
Supplement: Supplementary file 1 [file DataSheet1.docx]

**Contents**

[Table S1. Two-by-two contingency table for measure of disproportionality 2](#_Toc198561779)

[Table S2. Measure of disproportionality and signal generation criteria 3](#_Toc198561780)

[Table S3. Signal strength of the top fifty pneumococcal vaccine-related positive PT signals and their corresponding SOCs 4](#_Toc198561781)

[Table S4. Signal strength of DME screening results for pneumococcal vaccines 9](#_Toc198561782)

[Figure S1. The forest plot of pneumococcal vaccine-related positive signals matched PTs in the DME list and their corresponding SOCs 14](#_Toc198561783)

# Table S1. Two-by-two contingency table for measure of disproportionality

|  | No. target adverse reaction reports | No. other adverse reaction reports | Total |
| --- | --- | --- | --- |
| Target drug | a | b | a + b |
| Other drugs | c | d | c + d |
| Total | a + c | b + d | N = a + b + c + d |

a, number of reports containing both the suspect drug and the suspect adverse drug reaction; b, number of reports containing the suspect adverse drug reaction with other medications (except the drug of interest); c, number of reports containing the suspect drug with other adverse drug reactions (except the event of interest); d, number of reports containing other medications and other adverse drug reactions.

# Table S2. Measure of disproportionality and signal generation criteria

| Method | Formula | Threshold |
| --- | --- | --- |
| ROR | $ROR=\frac{a/c}{b/d}=\frac{ad}{bc}$  $SE(lnROR)=\sqrt{(\frac{1}{a}+\frac{1}{b}+\frac{1}{c}+\frac{1}{d})}$  $95\% CI=e^{ln\left( ROR \right)\pm1.96\sqrt{(\frac{1}{a}+\frac{1}{b}+\frac{1}{c}+\frac{1}{d})}}$ | a ≥ 3 and 95% CI (lower limit) > 1 |
| PRR | $PRR=\frac{a/{(a+b)}}{c/{(c+d)}}$  $95\% CI=e^{ln(PRR)\pm1.96\sqrt{(\frac{1}{a}-\frac{1}{a+b}+\frac{1}{c}-\frac{1}{c+d})}}$  ${}^{2}=\frac{{N(\left\vert ad-bc \right\vert-\frac{N}{2})}^{2}}{(a+b)(c+d)(b+d)(a+c)}$ | a ≥ 3 and PRR ≥ 2 and χ^2^ ≥ 4 |
| BCPNN | $IC={log}_{2}^{\frac{p(x,y)}{p\left( x \right)p(y)}}={log}_{2}^{\frac{a(a+b+c+d)}{(a+b)(a+c)}}$  $E\left( IC \right)={log}_{2}^{\frac{(a+\gamma ij)(a+b+c+d+\alpha)(a+b+c+d+\beta)}{(a+b+c+d+\gamma)(a+b+\alpha i)(a+b+\beta j)}}$  $V(IC)$=$\frac{1}{{(ln2)}^{2}}\left\{ \left[ \frac{\left( a+b+c+d \right)-a+\gamma-\gamma ij}{(a+\gamma ij)(1+a+b+c+d+\gamma)} \right]+\left[ \frac{\left( a+b+c+d \right)-(a+b)+\alpha-\alpha i}{(a+b+\alpha i)(1+a+b+c+d+\alpha)} \right]+\left[ \frac{\left( a+b+c+d \right)-(a+c)+\beta-\beta j}{(a+b+\beta j)(1+a+b+c+d+\beta)} \right] \right\}$  $\gamma=\gamma ij\frac{(a+b+c+d+\alpha)(a+b+c+d+\beta)}{(a+b+\alpha i)(a+c+\beta j)}$  *IC-2SD*$=E\left( IC \right)-2\sqrt{V(IC)}$ | a ≥ 3 and IC-2SD > 0 |
| EBGM | $EBGM=\frac{a(a+b+c+d)}{(a+c)(a+b)}$  $EBGM05=e^{\ln\left( EBGM \right)-1.64\sqrt{(\frac{1}{a}+\frac{1}{b}+\frac{1}{c}+\frac{1}{d})}}$ | a > 0 and EBGM05 > 2 |

ROR, reporting odds ratio; CI, confidence interval; PRR, proportional reporting ratio; BCPNN, Bayesian confidence propagation neural network; IC, information components; MGPS, multi-item gamma Poisson shrinker; EBGM, empirical Bayes geometric mean; EBGM05, the lower 95% one-side CI of EBGM; IC-2SD, the lower limit of the 95% two-sided CI of the IC; E is the expected number of cases.

# Table S3. Signal strength of the top fifty pneumococcal vaccine-related positive PT signals and their corresponding SOCs

| SOC | PT | a | ROR (95%Cl) | PRR (χ^2^) | EBGM (EBGM05) | IC (IC-2SD) | *p*-adjust |
| --- | --- | --- | --- | --- | --- | --- | --- |
| General disorders and administration site conditions | INJECTION SITE ERYTHEMA | 24675 | 4.24  (4.18 - 4.3) | 4.12  (47340.1) | 3.51  (3.46) | 1.81  (1.79) | ≤ 0.05 |
| General disorders and administration site conditions | INJECTION SITE SWELLING | 17938 | 4.19  (4.12 - 4.26) | 4.10  (34053.61) | 3.49  (3.44) | 1.80  (1.78) | ≤ 0.05 |
| General disorders and administration site conditions | INJECTION SITE PAIN | 17827 | 2.75  (2.71 - 2.8) | 2.7  (16691.77) | 2.47  (2.44) | 1.30  (1.28) | ≤ 0.05 |
| Skin and subcutaneous tissue disorders | ERYTHEMA | 17778 | 3.99  (3.92 - 4.06) | 3.9  (31464.76) | 3.36  (3.31) | 1.75  (1.72) | ≤ 0.05 |
| General disorders and administration site conditions | INJECTION SITE WARMTH | 9224 | 3.63  (3.55 - 3.71) | 3.59  (14284.71) | 3.14  (3.08) | 1.65  (1.62) | ≤ 0.05 |
| General disorders and administration site conditions | CRYING | 6389 | 8.28  (8.04 - 8.53) | 8.21  (27253.46) | 5.85  (5.70) | 2.55  (2.51) | ≤ 0.05 |
| General disorders and administration site conditions | PERIPHERAL SWELLING | 6291 | 2.68  (2.61 - 2.75) | 2.66  (5665.49) | 2.44  (2.38) | 1.28  (1.25) | ≤ 0.05 |
| General disorders and administration site conditions | SWELLING | 5959 | 2.80  (2.72 - 2.88) | 2.78  (5856.05) | 2.53  (2.47) | 1.34  (1.30) | ≤ 0.05 |
| Skin and subcutaneous tissue disorders | SKIN WARM | 5701 | 4.19  (4.07 - 4.31) | 4.16  (11010.89) | 3.54  (3.45) | 1.82  (1.78) | ≤ 0.05 |
| Infections and infestations | CELLULITIS | 5521 | 8.02  (7.77 - 8.28) | 7.96  (22860.74) | 5.73  (5.58) | 2.52  (2.47) | ≤ 0.05 |
| Psychiatric disorders | IRRITABILITY | 4920 | 11.06  (10.67 - 11.46) | 10.98  (27066.53) | 7.05  (6.84) | 2.82  (2.77) | ≤ 0.05 |
| General disorders and administration site conditions | OEDEMA PERIPHERAL | 4663 | 4.59  (4.44 - 4.74) | 4.56  (10227.43) | 3.80  (3.70) | 1.93  (1.88) | ≤ 0.05 |
| General disorders and administration site conditions | INJECTION SITE OEDEMA | 4662 | 4.39  (4.25 - 4.54) | 4.37  (9635.21) | 3.68  (3.58) | 1.88  (1.83) | ≤ 0.05 |
| Investigations | STREPTOCOCCUS TEST POSITIVE | 4238 | 140.11  (127.85 - 153.55) | 139.18  (62912.17) | 15.95  (14.77) | 4.00  (3.93) | ≤ 0.05 |
| General disorders and administration site conditions | INJECTION SITE REACTION | 3867 | 3.40  (3.28 - 3.52) | 3.38  (5413.76) | 2.98  (2.90) | 1.58  (1.53) | ≤ 0.05 |
| General disorders and administration site conditions | INJECTION SITE INDURATION | 3799 | 3.41  (3.29 - 3.53) | 3.39  (5352.48) | 2.99  (2.91) | 1.58  (1.53) | ≤ 0.05 |
| Investigations | BODY TEMPERATURE INCREASED | 3760 | 3.56  (3.44 - 3.69) | 3.55  (5693.34) | 3.10  (3.01) | 1.63  (1.58) | ≤ 0.05 |
| Infections and infestations | PNEUMONIA PNEUMOCOCCAL | 3570 | 1010.05  (782.58 - 1303.65) | 1004.35  (59166.25) | 17.58  (14.2) | 4.14  (4.07) | ≤ 0.05 |
| Vascular disorders | PALLOR | 3139 | 2.26  (2.18 - 2.35) | 2.25  (1937.25) | 2.11  (2.04) | 1.07  (1.02) | ≤ 0.05 |
| General disorders and administration site conditions | INJECTION SITE RASH | 2808 | 2.25  (2.17 - 2.34) | 2.25  (1721.68) | 2.10  (2.03) | 1.07  (1.01) | ≤ 0.05 |
| Infections and infestations | PNEUMONIA | 2773 | 3.74  (3.59 - 3.90) | 3.73  (4546.95) | 3.24  (3.13) | 1.69  (1.64) | ≤ 0.05 |
| General disorders and administration site conditions | INJECTED LIMB MOBILITY DECREASED | 2713 | 4.81  (4.61 - 5.02) | 4.79  (6344.71) | 3.95  (3.81) | 1.98  (1.92) | ≤ 0.05 |
| Nervous system disorders | HYPOTONIA | 2667 | 6.40  (6.12 - 6.69) | 6.38  (8787.19) | 4.90  (4.72) | 2.29  (2.23) | ≤ 0.05 |
| General disorders and administration site conditions | TENDERNESS | 2570 | 3.08  (2.95 - 3.21) | 3.07  (3033.59) | 2.75  (2.65) | 1.46  (1.40) | ≤ 0.05 |
| General disorders and administration site conditions | INJECTION SITE HYPERSENSITIVITY | 2549 | 2.76  (2.64 - 2.88) | 2.75  (2445.90) | 2.51  (2.42) | 1.32  (1.26) | ≤ 0.05 |
| Nervous system disorders | CONVULSION | 2512 | 3.69  (3.53 - 3.85) | 3.68  (4028.79) | 3.20  (3.09) | 1.68  (1.62) | ≤ 0.05 |
| Investigations | WHITE BLOOD CELL COUNT INCREASED | 2400 | 6.55  (6.25 - 6.87) | 6.53  (8111.87) | 4.99  (4.79) | 2.32  (2.25) | ≤ 0.05 |
| General disorders and administration site conditions | SCREAMING | 2134 | 4.87  (4.64 - 5.11) | 4.85  (5074.21) | 3.99  (3.83) | 2.00  (1.93) | ≤ 0.05 |
| Nervous system disorders | FEBRILE CONVULSION | 1955 | 6.55  (6.22 - 6.91) | 6.54  (6613.74) | 4.99  (4.78) | 2.32  (2.25) | ≤ 0.05 |
| Vascular disorders | CYANOSIS | 1952 | 5.64  (5.36 - 5.94) | 5.63  (5574.56) | 4.47  (4.28) | 2.16  (2.09) | ≤ 0.05 |
| General disorders and administration site conditions | INJECTION SITE MASS | 1924 | 2.37  (2.26 - 2.49) | 2.37  (1332.57) | 2.20  (2.11) | 1.14  (1.07) | ≤ 0.05 |
| Investigations | C-REACTIVE PROTEIN INCREASED | 1759 | 3.67  (3.48 - 3.86) | 3.66  (2796.77) | 3.19  (3.05) | 1.67  (1.60) | ≤ 0.05 |
| General disorders and administration site conditions | OEDEMA | 1731 | 3.32  (3.16 - 3.50) | 3.32  (2343.61) | 2.94  (2.81) | 1.55  (1.48) | ≤ 0.05 |
| Gastrointestinal disorders | INTUSSUSCEPTION | 1628 | 17.99  (16.78 - 19.29) | 17.95  (12631.63) | 9.21  (8.69) | 3.20  (3.12) | ≤ 0.05 |
| Gastrointestinal disorders | HAEMATOCHEZIA | 1605 | 7.02  (6.62 - 7.44) | 7.00  (5839.58) | 5.24  (4.99) | 2.39  (2.31) | ≤ 0.05 |
| Infections and infestations | PNEUMOCOCCAL BACTERAEMIA | 1567 | 1657.28  (1012.76 - 2711.98) | 1653.18  (26155.46) | 17.70  (11.72) | 4.15  (4.04) | ≤ 0.05 |
| Skin and subcutaneous tissue disorders | RASH MACULAR | 1531 | 2.47  (2.34 - 2.61) | 2.47  (1166.89) | 2.28  (2.18) | 1.19  (1.11) | ≤ 0.05 |
| General disorders and administration site conditions | LOCAL REACTION | 1424 | 4.63  (4.37 - 4.91) | 4.62  (3175.32) | 3.84  (3.66) | 1.94  (1.86) | ≤ 0.05 |
| Nervous system disorders | UNRESPONSIVE TO STIMULI | 1421 | 2.44  (2.31 - 2.58) | 2.44  (1051.42) | 2.25  (2.15) | 1.17  (1.09) | ≤ 0.05 |
| Infections and infestations | INJECTION SITE CELLULITIS | 1398 | 8.82  (8.27 - 9.41) | 8.81  (6358.93) | 6.13  (5.81) | 2.62  (2.53) | ≤ 0.05 |
| General disorders and administration site conditions | INDURATION | 1382 | 3.44  (3.24 - 3.64) | 3.43  (1978.89) | 3.02  (2.88) | 1.59  (1.51) | ≤ 0.05 |
| Respiratory, thoracic and mediastinal disorders | APNOEA | 1322 | 7.89  (7.39 - 8.43) | 7.88  (5415.58) | 5.69  (5.39) | 2.51  (2.42) | ≤ 0.05 |
| General disorders and administration site conditions | LOCAL SWELLING | 1305 | 7.62  (7.14 - 8.13) | 7.60  (5162.06) | 5.55  (5.26) | 2.47  (2.38) | ≤ 0.05 |
| Skin and subcutaneous tissue disorders | RASH GENERALISED | 1241 | 3.99  (3.75 - 4.24) | 3.98  (2242.79) | 3.41  (3.24) | 1.77  (1.68) | ≤ 0.05 |
| General disorders and administration site conditions | INJECTION SITE INFLAMMATION | 1191 | 4.21  (3.95 - 4.49) | 4.21  (2331.07) | 3.57  (3.38) | 1.83  (1.74) | ≤ 0.05 |
| Nervous system disorders | HYPOTONIC-HYPORESPONSIVE EPISODE | 1147 | 10.80  (10.03 - 11.63) | 10.78  (6212.43) | 6.97  (6.55) | 2.80  (2.7) | ≤ 0.05 |
| Investigations | LABORATORY TEST ABNORMAL | 1112 | 2.38  (2.24 - 2.54) | 2.38  (779.71) | 2.21  (2.10) | 1.14  (1.05) | ≤ 0.05 |
| Nervous system disorders | HYPOKINESIA | 996 | 2.52  (2.36 - 2.69) | 2.52  (791.85) | 2.32  (2.19) | 1.21  (1.12) | ≤ 0.05 |
| Infections and infestations | PNEUMOCOCCAL INFECTION | 992 | 349.40  (261.53 - 466.79) | 348.85  (15882.05) | 17.05  (13.38) | 4.09  (3.96) | ≤ 0.05 |
| Nervous system disorders | DYSKINESIA | 974 | 3.02  (2.83 - 3.24) | 3.02  (1118.07) | 2.71  (2.56) | 1.44  (1.34) | ≤ 0.05 |

PT, preferred term; SOC, System Organ Class; CI, confidence interval; ROR, reporting odds ratio; PRR, proportional reporting ratio; χ^2^, Chi-squared; IC, information component; IC-2SD, the lower limit of the 95% two-sided CI of the IC; EBGM, empirical Bayesian geometric mean; EBGM05, the lower 95 two-sided CI of EBGM.

# Table S4. Signal strength of DME screening results for pneumococcal vaccines

| SOC | PT | a | ROR (95%Cl) | PRR (χ^2^) | EBGM (EBGM05) | IC (IC-2SD) |
| --- | --- | --- | --- | --- | --- | --- |
| Immune system disorders | ANAPHYLACTIC REACTION | 415 | 0.58  (0.53, 0.64) | 0.58  (121.47) | 0.59  (0.55) | -0.75  (-0.89) |
| Skin and subcutaneous tissue disorders | ERYTHEMA MULTIFORME | 398 | 2.38  (2.14, 2.65) | 2.38  (279.60) | 2.21  (2.02) | 1.14  (0.99) |
| Skin and subcutaneous tissue disorders | ANGIOEDEMA | 190 | 0.58  (0.5, 0.67) | 0.58  (55.91) | 0.59  (0.53) | -0.75  (-0.96) |
| Immune system disorders | ANAPHYLACTIC SHOCK | 141 | 1.10  (0.93, 1.31) | 1.10  (1.24) | 1.10  (0.95) | 0.13  (-0.12) |
| Blood and lymphatic system disorders | IMMUNE THROMBOCYTOPENIA | 116 | 0.97  (0.81, 1.17) | 0.97  (0.08) | 0.97  (0.83) | -0.04  (-0.31) |
| Renal and urinary disorders | RENAL FAILURE | 107 | 0.96  (0.79, 1.17) | 0.96  (0.14) | 0.97  (0.82) | -0.05  (-0.34) |
| Ear and labyrinth disorders | DEAFNESS | 90 | 0.31  (0.25, 0.38) | 0.31  (134.27) | 0.32  (0.27) | -1.62  (-1.93) |
| Renal and urinary disorders | ACUTE KIDNEY INJURY | 82 | 0.30  (0.24, 0.38) | 0.30  (129.79) | 0.31  (0.26) | -1.67  (-1.99) |
| Eye disorders | BLINDNESS | 60 | 0.27  (0.21, 0.35) | 0.27  (114.96) | 0.28  (0.23) | -1.82  (-2.19) |
| Immune system disorders | ANAPHYLACTOID REACTION | 50 | 1.14  (0.85, 1.51) | 1.14  (0.76) | 1.13  (0.89) | 0.17  (-0.24) |
| Musculoskeletal and connective tissue disorders | RHABDOMYOLYSIS | 49 | 0.79  (0.59, 1.05) | 0.79  (2.64) | 0.80  (0.63) | -0.32  (-0.74) |
| Blood and lymphatic system disorders | HAEMOLYTIC ANAEMIA | 45 | 2.12  (1.55, 2.88) | 2.12  (23.53) | 1.99  (1.54) | 0.99  (0.54) |
| Respiratory, thoracic and mediastinal disorders | PULMONARY FIBROSIS | 36 | 1.26  (0.89, 1.76) | 1.26  (1.74) | 1.24  (0.93) | 0.31  (-0.18) |
| Blood and lymphatic system disorders | PANCYTOPENIA | 32 | 0.90  (0.63, 1.28) | 0.90  (0.36) | 0.90  (0.67) | -0.15  (-0.66) |
| Blood and lymphatic system disorders | HAEMOLYSIS | 32 | 1.76  (1.22, 2.53) | 1.76  (9.5) | 1.69  (1.24) | 0.76  (0.23) |
| Skin and subcutaneous tissue disorders | STEVENS-JOHNSON SYNDROME | 31 | 1.30  (0.9, 1.87) | 1.30  (1.94) | 1.27  (0.94) | 0.35  (-0.18) |
| Blood and lymphatic system disorders | AUTOIMMUNE HAEMOLYTIC ANAEMIA | 28 | 1.44  (0.98, 2.12) | 1.44  (3.48) | 1.41  (1.02) | 0.49  (-0.07) |
| Hepatobiliary disorders | HEPATIC FAILURE | 27 | 1.31  (0.89, 1.94) | 1.31  (1.87) | 1.29  (0.93) | 0.37  (-0.20) |
| Gastrointestinal disorders | INTESTINAL PERFORATION | 26 | 3.99  (2.6, 6.12) | 3.99  (47.12) | 3.42  (2.39) | 1.77  (1.17) |
| Ear and labyrinth disorders | SUDDEN HEARING LOSS | 23 | 0.19  (0.12, 0.28) | 0.19  (79.79) | 0.20  (0.14) | -2.34  (-2.94) |
| Blood and lymphatic system disorders | AGRANULOCYTOSIS | 16 | 3.33  (1.95, 5.7) | 3.33  (21.83) | 2.95  (1.88) | 1.56  (0.8) |
| Blood and lymphatic system disorders | APLASTIC ANAEMIA | 14 | 0.96  (0.56, 1.65) | 0.96  (0.02) | 0.96  (0.61) | -0.05  (-0.82) |
| Respiratory, thoracic and mediastinal disorders | PULMONARY HYPERTENSION | 14 | 0.45  (0.26, 0.76) | 0.45  (9.39) | 0.46  (0.30) | -1.12  (-1.88) |
| Gastrointestinal disorders | PANCREATITIS | 13 | 0.24  (0.14, 0.42) | 0.24  (30.72) | 0.25  (0.16) | -1.99  (-2.77) |
| Ear and labyrinth disorders | DEAFNESS NEUROSENSORY | 12 | 0.22  (0.12, 0.38) | 0.22  (33.56) | 0.23  (0.14) | -2.14  (-2.95) |
| Skin and subcutaneous tissue disorders | DERMATITIS EXFOLIATIVE | 11 | 0.81  (0.44, 1.48) | 0.81  (0.46) | 0.82  (0.49) | -0.29  (-1.14) |
| Cardiac disorders | VENTRICULAR FIBRILLATION | 10 | 0.26  (0.14, 0.48) | 0.26  (20.79) | 0.27  (0.16) | -1.88  (-2.76) |
| Skin and subcutaneous tissue disorders | DERMATITIS EXFOLIATIVE GENERALISED | 10 | 1.02  (0.54, 1.94) | 1.02  (0.00) | 1.02  (0.6) | 0.03  (-0.87) |
| Renal and urinary disorders | AZOTAEMIA | 9 | 1.08  (0.55, 2.11) | 1.08  (0.05) | 1.07  (0.61) | 0.10  (-0.84) |
| Blood and lymphatic system disorders | THROMBOTIC THROMBOCYTOPENIC PURPURA | 9 | 0.53  (0.27, 1.04) | 0.53  (3.57) | 0.55  (0.31) | -0.87  (-1.8) |
| Hepatobiliary disorders | AUTOIMMUNE HEPATITIS | 9 | 0.25  (0.13, 0.48) | 0.25  (20.19) | 0.26  (0.15) | -1.95  (-2.87) |
| Skin and subcutaneous tissue disorders | DRUG REACTION WITH EOSINOPHILIA AND SYSTEMIC SYMPTOMS | 8 | 1.15  (0.56, 2.36) | 1.15  (0.15) | 1.14  (0.63) | 0.19  (-0.8) |
| Blood and lymphatic system disorders | GRANULOCYTOPENIA | 7 | 3.94  (1.73, 8.97) | 3.94  (12.44) | 3.38  (1.7) | 1.76  (0.64) |
| Blood and lymphatic system disorders | BONE MARROW FAILURE | 6 | 1.32  (0.57, 3.02) | 1.32  (0.42) | 1.29  (0.64) | 0.37  (-0.76) |
| Blood and lymphatic system disorders | FEBRILE NEUTROPENIA | 6 | 0.77  (0.34, 1.74) | 0.77  (0.41) | 0.78  (0.39) | -0.36  (-1.48) |
| Hepatobiliary disorders | DRUG-INDUCED LIVER INJURY | 5 | 0.66  (0.27, 1.62) | 0.66  (0.81) | 0.68  (0.32) | -0.56  (-1.77) |
| Gastrointestinal disorders | PANCREATITIS ACUTE | 5 | 0.12  (0.05, 0.28) | 0.12  (33.66) | 0.12  (0.06) | -3.04  (-4.22) |
| Ear and labyrinth disorders | DEAFNESS TRANSITORY | 5 | 0.47  (0.19, 1.15) | 0.47  (2.88) | 0.49  (0.23) | -1.04  (-2.24) |
| Hepatobiliary disorders | ACUTE HEPATIC FAILURE | 5 | 0.60  (0.25, 1.46) | 0.60  (1.30) | 0.61  (0.29) | -0.71  (-1.91) |
| Blood and lymphatic system disorders | APLASIA PURE RED CELL | 4 | 3.75  (1.27, 11.08) | 3.75  (6.60) | 3.25  (1.31) | 1.7  (0.28) |
| Product issues | PRODUCT CONTAMINATION MICROBIAL | 4 | 11.25  (3.18, 39.88) | 11.25  (22.42) | 7.15  (2.48) | 2.84  (1.28) |
| Skin and subcutaneous tissue disorders | TOXIC EPIDERMAL NECROLYSIS | 4 | 0.77  (0.28, 2.09) | 0.77  (0.27) | 0.78  (0.34) | -0.36  (-1.69) |
| Hepatobiliary disorders | HEPATIC NECROSIS | 3 | 0.97  (0.3, 3.12) | 0.97  (0.00) | 0.98  (0.37) | -0.04  (-1.53) |
| Hepatobiliary disorders | HEPATITIS FULMINANT | 3 | 1.95  (0.59, 6.44) | 1.95  (1.24) | 1.85  (0.68) | 0.89  (-0.65) |
| Ear and labyrinth disorders | DEAFNESS PERMANENT | 2 | 0.69  (0.17, 2.83) | 0.69  (0.27) | 0.70  (0.21) | -0.51  (-2.23) |
| General disorders and administration site conditions | SUDDEN CARDIAC DEATH | 2 | 0.18  (0.05, 0.73) | 0.18  (7.3) | 0.19  (0.06) | -2.39  (-4.07) |
| Cardiac disorders | TORSADE DE POINTES | 1 | 0.51  (0.07, 3.74) | 0.51  (0.45) | 0.53  (0.1) | -0.93  (-3.02) |
| Gastrointestinal disorders | OEDEMATOUS PANCREATITIS | 1 | 0.89  (0.12, 6.64) | 0.89  (0.01) | 0.89  (0.17) | -0.16  (-2.30) |
| Eye disorders | OPTIC ISCHAEMIC NEUROPATHY | 1 | 0.05  (0.01, 0.36) | 0.05  (18.02) | 0.05  (0.01) | -4.24  (-6.29) |
| Immune system disorders | ANAPHYLACTOID SHOCK | 1 | 2.41  (0.30, 19.60) | 2.41  (0.72) | 2.23  (0.39) | 1.16  (-1.10) |
| Respiratory, thoracic and mediastinal disorders | PULMONARY ARTERIAL HYPERTENSION | 1 | 0.21  (0.03, 1.48) | 0.21  (3.03) | 0.22  (0.04) | -2.21  (-4.28) |

DME, designated medical events; PT, preferred term; SOC, System Organ Class; CI, confidence interval; ROR, reporting odds ratio; PRR, proportional reporting ratio; χ^2^, Chi-squared; IC, information component; IC-2SD, the lower limit of the 95% two-sided CI of the IC; EBGM, empirical Bayesian geometric mean; EBGM05, the lower 95 two-sided CI of EBGM.





# Figure S1. The forest plot of pneumococcal vaccine-related positive signals matched PTs in the DME list and their corresponding SOCs

DME, designated medical events. PTs, preferred terms; SOCs, system organ classes.
